# Case Report: A Case of Pituitary Carcinoma Treated With Sequential Dual Immunotherapy and Vascular Endothelial Growth Factor Inhibition Therapy

**DOI:** 10.3389/fendo.2020.576027

**Published:** 2020-11-18

**Authors:** Lydia S. Lamb, Hao-Wen Sim, Ann I. McCormack

**Affiliations:** ^1^ Garvan Institute of Medical Research, Sydney, NSW, Australia; ^2^ St Vincent’s Clinical School, University of New South Wales, Sydney, NSW, Australia; ^3^ The Kinghorn Cancer Centre, Sydney, NSW, Australia; ^4^ Department of Endocrinology, St Vincent’s Hospital, Sydney, NSW, Australia

**Keywords:** ****aggressive pituitary tumors, pituitary carcinoma, immunotherapy, immune checkpoint inhibitor, vascular endothelial growth factor inhibitor, biomarker, combination therapy

## Abstract

Aggressive pituitary tumors (APTs) are associated with significant morbidity and mortality, and effective treatment options are limited. Immune checkpoint inhibitors (ICIs) have revolutionized clinical cancer care; however, there is little experience with these agents in the management of APTs. Vascular endothelial growth factor (VEGF) targeted therapy has reported success in a small number of APT case reports. Here we describe a case of pituitary carcinoma responding to ICI therapy and subsequently VEGF inhibition. We discuss the possible mechanisms and experience with ICI therapy and VEGF inhibitors in the management of APTs, biomarkers that may predict response, and the potential role of combination therapies including ICIs and temozolomide.

## Introduction

Aggressive pituitary tumors (APTs) including pituitary carcinomas (PCs) are a subset of pituitary tumors with a more aggressive course defined clinically by significant invasion, rapid tumor growth rate, and resistance to optimal standard therapies (1). Morbidity and mortality associated with these tumors are high with overall mortality rates for APT and PC reported at 28 and 42.5% respectively at a median duration of 11 years following diagnosis (2). Treatment options include a combination of surgical resection, radiotherapy and medical therapy for functioning tumors. Temozolomide (TMZ) is the only chemotherapeutic agent demonstrated to have a significant effect on APTs and is recommended as first line chemotherapy for APTs in the European Society of Endocrinology (ESE) Clinical Practice Guidelines (3). TMZ is associated with an improved 5 year overall survival in APTs of up to 90% in responders; however, only 37% of patients have a response to TMZ. One third of patients continue to progress despite treatment, and 35% initially responding to TMZ progress following cessation of treatment (2–4). There is typically a poor response to a second course of TMZ with just 11% of patients reported to have partial regression following a second course in the ESE survey (2). Alternative treatment options remain experimental and include use of targeted therapies such as VEGF, mTOR, or EGFR inhibitors and peptide receptor radionucleotide therapy (5).

Immune checkpoint inhibitors (ICIs) have revolutionized clinical cancer care and are now approved for use in many malignancies including melanoma, lung cancer, renal cell carcinoma, squamous cell, head and neck cancer, lymphoma, urothelial carcinoma and gastro-esophageal carcinoma (6). ICIs are monoclonal antibodies targeting the immunosuppressive CTLA4 and PD-1 receptors on the surface of T lymphocytes and PD-L1 on the surface of tumor cells, inhibiting the downregulation of T lymphocytes and resulting in improved anti-tumor immune response.

Thirteen cases of APTs treated with VEGF inhibition therapy have been reported, with the majority responding to treatment (2, 7–12). Published experience with ICI therapy in APTs is limited to two cases with markedly different outcomes (13, 14). No cases to our knowledge have previously been reported of treatment with ICI and VEGF inhibition therapy. Here we describe a case of PC responding to ICI treatment and subsequently VEGF inhibition. We discuss mechanisms supporting the use of ICIs in APTs and explore the potential for sequential and combination therapy of novel agents in the management of APTs.

## Case Description

**Figure 1:**
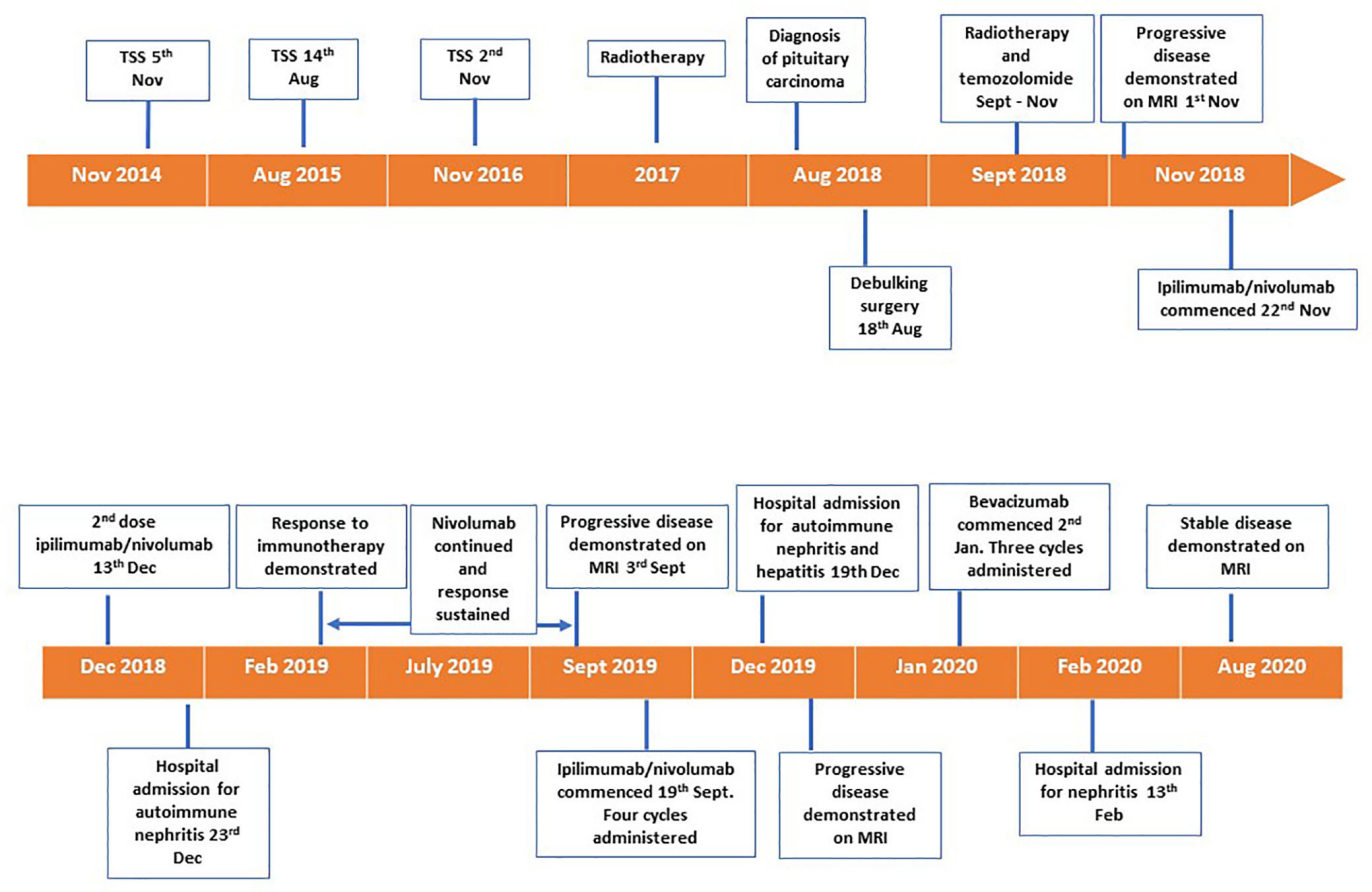
Timeline of disease progression and treatment.

**Figure 2:**
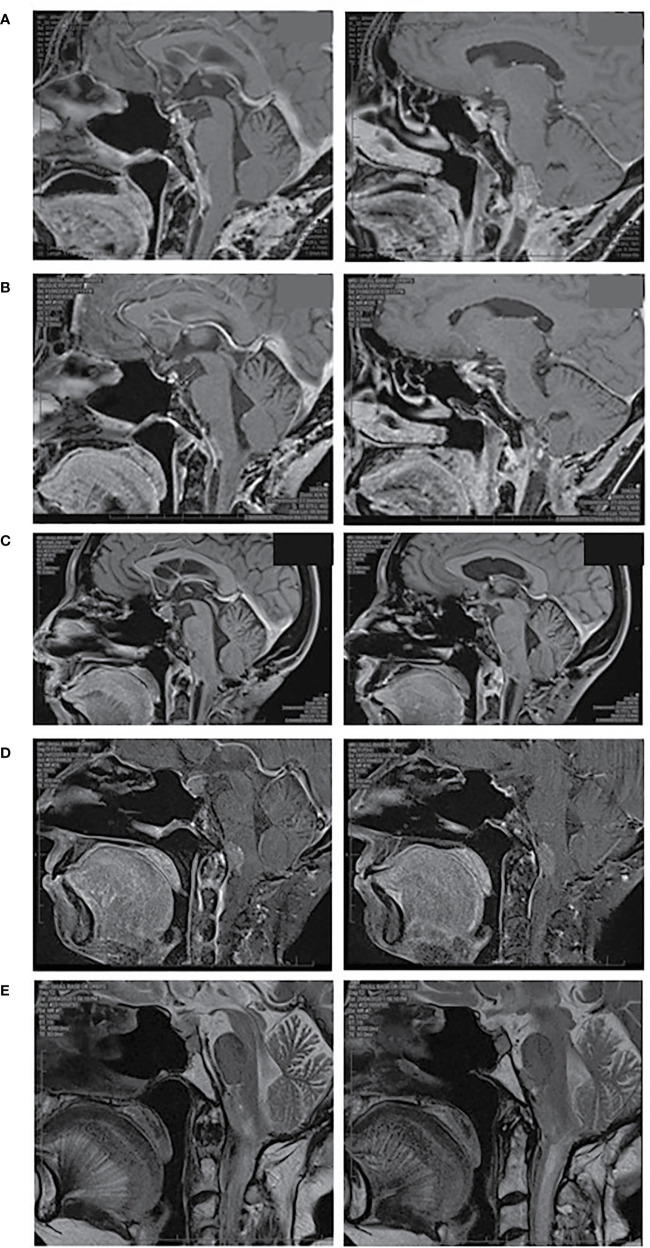
MRI pituitary, sagittal views. **(A)** October 2018 prior to dual ICI therapy, **(B)** June 2019 demonstrating response to dual ICI therapy, **(C)** September 2019 demonstrating progressive disease, **(D)** December 2019 demonstrating progressive disease following repeat dual ICI therapy, **(E)** April 2020 demonstrating stable disease following bevacizumab. **(A–D)** T1 weighted images post gadolinium, **(E)** T2 weighted images without gadolinium due to renal impairment.

**Figure 3:**
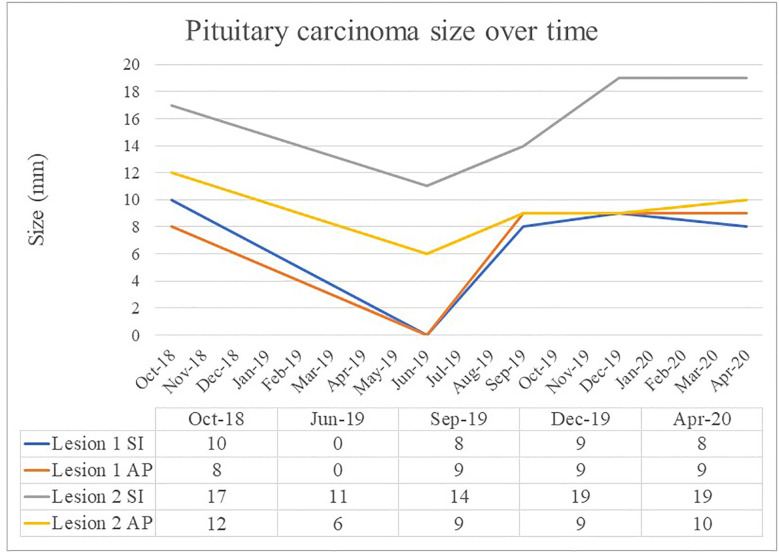
Pituitary carcinoma size (mm) over time. Superior to inferior measurement (SI), Anterior to posterior measurement (AP).

A 72 year old female was diagnosed with a silent lactotroph pituitary carcinoma in August 2018 (**Figure 1**). She underwent an initial transsphenoidal surgery (TSS) in 2014 following presentation with headaches and demonstration of a macroadenoma on MRI. Pituitary hormone profile was normal, and specifically she has never had hyperprolactinemia. Histopathology (2014) revealed a pituitary tumor with diffusely strong prolactin immunoexpression, mitoses 5 per 10 high power field and elevated Ki67 10%. Gross total resection was achieved at the initial surgery, however; she had subsequent rapid tumor recurrences necessitating repeat TSS in 2015 and 2016 followed by sellar radiotherapy in 2017. In 2018 the patient represented with headache, anorexia, and weight loss and MRI demonstrated extra-sellar disease progression with evidence of dural metastases. She underwent debulking surgery of the spinal metastatic disease in August 2018. Histopathology was consistent with a lactotroph (Pit-1 positive) pituitary carcinoma with high MGMT expression and Ki67 20%. On the recommendation of recent guidelines, albeit noting the high MGMT expression, she commenced a trial of TMZ, but progression was expected and demonstrated following 3 months of therapy (3). She also received concomitant radiotherapy delivered to the sites of spinal metastatic disease. In the meantime, tumor genomic profiling was undertaken. The tumor was mismatch repair-proficient, harbored no actionable variants, and the tumor mutation burden was low at 6.8 Mut/Mb. Tumor PD-L1 expression by immunohistochemistry was <1%. Beyond the use of temozolomide as first line chemotherapy for APT and PC, treatment options remain experimental and were presented to the patient. Extrapolating from the isolated case report of successful response to dual immunotherapy in an ACTH-secreting hypermutated pituitary carcinoma (13), as well as increasing use of immunotherapy in the cancer field, our patient elected to receive self-funded dual immunotherapy with ipilimumab 3 mg/kg IV and nivolumab 1 mg/kg IV thrice weekly. Following the second cycle, treatment was complicated by autoimmune nephritis with acute kidney injury requiring hospital admission. Creatinine peaked at 468 umol/L, eGFR 8 ml/min/1.73 m^2^. This responded to high dose glucocorticoid therapy with recovery over 10 days. Ipilimumab was discontinued and maintenance nivolumab 3 mg/kg IV twice weekly was continued for 17 cycles over 8 months. There was a marked clinical and radiological response of the primary pituitary carcinoma and all metastatic lesions that was sustained for eight months (**Figures 2** and **3**). In September 2019 tumor progression was again demonstrated despite continued use of nivolumab, and a decision was made to rechallenge with addition of ipilimumab to nivolumab. Following four cycles of combination ipilimumab and nivolumab, treatment was once again complicated by ICI-related nephritis and hepatitis which responded to high dose glucocorticoid therapy. Unfortunately, on this occasion MRI demonstrated tumor progression, and ipilimumab and nivolumab were ceased. Based on a small selection of successful case reports (2, 7–10, 12), further treatment was trialed with the VEGF monoclonal antibody bevacizumab 10 mg/kg IV twice weekly, and three cycles were administered. Treatment was interrupted by nephritis, and she was managed for both potential autoimmune and VEGF inhibitor induced nephritis with good response to high dose glucocorticoid therapy and antihypertensives. Six months after ceasing bevacizumab, she has stable disease on MRI.

## Discussion

To our knowledge, this is the first case reported of an APT treated with sequential ICI and VEGF targeted therapy. ICIs are now well established as effective treatments in a number of cancers; however, only two cases of use in APTs have previously been described. The case we have described highlights a number of interesting and relevant considerations for ICI and VEGF inhibitor use in the management of APTs.

### Immune Checkpoint Inhibitors and Normal Pituitary

The well-documented side effect of hypophysitis resulting from ICI treatment of other cancers provides a rationale for the use of these agents in the management of pituitary tumors. A recent meta-analysis reported the incidence of hypophysitis following ICI therapy was greatest with combination therapy (6.4%) and was significantly greater in those receiving CTLA-4 inhibitors (3.2%) compared with PD-1 inhibitors (0.4%) (OR, 0.29; 95% CI, 0.18–0.49; P <.001) (15). The mechanism of action of CTLA4 inhibitors on the pituitary seems to be multifactorial. Pituitary antibodies have been demonstrated in CTLA-4 inhibitor related hypophysitis (16). Pituitary expression of CTLA-4 has been demonstrated in normal pituitary and pituitary adenomas and may provide a direct target for CTLA4 antibodies (16, 17). Pituitary specific complement deposition in mice administered CTLA-4 antibody suggests that CTLA-4 monoclonal antibodies induce a type 2 hypersensitivity reaction which targets CTLA-4 expressed on pituitary cells and initiates tissue destruction (16). Direct binding of CTLA-4 antibody to anterior pituitary cells may also activate antibody dependent cell-mediated cytotoxicity (ADCC). By contrast, the IgG4-based PD-1 and PD-L1 antibodies do not activate the classical complement pathway and are less effective in activating ADCC (17). Despite this, treatments targeting PD-1 or PD-L1 remain intriguing as a potential treatment option for pituitary tumors based on the findings that pituitary tumors express variable levels of PD-L1 with increased expression in functioning tumors and an association with higher tumor Ki67 index (18–20).

### Clinical Experience With Immune Checkpoint Inhibitors in Aggressive Pituitary Tumors

Clinical experience with ICIs to treat APTs is limited with just two other cases reported in the literature to date. Lin et al. described the case of a 35 year old female with an aggressive ACTH secreting pituitary tumor that initially responded to combination TMZ and capecitabine prior to metastasizing to the liver. She subsequently received dual ICI therapy with ipilimumab and nivolumab resulting in a 92% reduction in volume of the liver metastasis, 59% reduction in intracranial tumor volume, and normalization of ACTH levels. The liver metastasis had low (<1%) PD-L1 expression by immunohistochemistry (13). Interestingly, genetic analysis of the hepatic metastasis demonstrated development of a hypermutated phenotype (5,275 mutations or 93 mutations/Mb) classic for TMZ exposure including an MSH6 mutation. TMZ is known to induce inactivating mutations in MSH6 in malignant gliomas which confers resistance to TMZ, and there is at least one other case of a PC with development of MSH6 deficiency and progression following TMZ (21–24). Caccese et al. reported the case of a 47 year old male with a silent corticotroph adenoma which transformed to an aggressive ACTH secreting tumor with progression despite surgery, radiotherapy, and TMZ. Immunohistochemical analysis following TMZ demonstrated complete loss of MSH2 and MSH6, and PD-L1 expression was 0%. The patient received four cycles of pembrolizumab; however, he continued to have radiological and biochemical disease progression (14).

### Combination Immune Checkpoint Inhibitor Therapy

It is possible that the excellent response to therapy observed in our case and that of Lin et al. was due to the use of combination CTLA4 and PD-L1 inhibition, compared with the use of pembrolizumab monotherapy as reported by Caccese et al. Pre-clinical studies suggest improved response to ICI combination therapy when compared to monotherapy by acting synergistically, increasing the number of tumor infiltrating lymphocytes, reducing T regulatory (T reg) lymphocytes, and retarding tumor growth (25, 26). Improved overall response and survival have been demonstrated with combination therapy compared with monotherapy for the treatment of melanoma and other cancers, albeit with an increased rate of autoimmune adverse effects (27–31).

### Biomarkers Predicting Response to Immune Checkpoint Inhibitor Therapy

Several potential biomarkers for response to ICI therapy have been proposed in the management of other cancers which can be considered in the context of the cases described. High PD-L1 expression on tumor cells has been associated with improved response rate and survival in a number of cancers treated with PD-L1/PD1 inhibition including melanoma, non-small cell lung cancer, urothelial cancer, and renal cell carcinoma (32–38). However, other studies have demonstrated no association between PD-L1 expression and response to treatment, and patients with PD-L1 negative disease can still achieve clinical benefit from PD-L1/PD1 inhibition (27, 39–41). Several reasons for the conflicting findings have been proposed. There is significant variability in the scoring systems for PD-L1 expression as well as the immunohistochemistry antibodies and platforms used in clinical trials (32, 33). Other factors that may account for PD-L1 variation include T cell derived cytokines, intracellular signaling pathways and transcription factors which upregulate PD-L1 expression (42). Furthermore, PD-L1 expression can be transient and variable depending on factors relating to treatment and cancer progression, and intratumoral heterogeneity may exist (43–45). These factors all contribute to the poor reliability of PD-L1 as a biomarker for response to ICI therapy. Our case as well as the other two published pituitary cases had low PD-L1 expression, yet two of the three cases demonstrated response to ICI therapy.

Elevated tumor mutational burden (TMB) has emerged as a predictor of improved survival and response to ICI in tumors such as melanoma and non-small cell lung cancer. It is thought that tumors with a high mutation burden express higher numbers of neoantigens that can be recognized by the immune system in response to immune checkpoint inhibitor therapy (46–48). A greater benefit with anti-PD-L1 and PD-1 therapy has been reported in tumors with both high TMB and high PD-L1 expression (46). However, response to combination ICI therapy with both PD-L1/PD-1 and CTLA4 blockade seems to be predicted by TMB but not PD-L1 expression (46).

In addition to TMB, improved survival and response to ICI is associated with mismatch repair deficiency (dMMR) which is defined by defects in one of four key genes (*MLH1*, *PMS2*, *MSH2*, and *MSH6*) that encode the MMR complex (48, 49). dMMR induces microsatellite instability (MSI) and results in a deficiency of DNA repair mechanisms, increased TMB, and associated neoantigens (48–50). dMMR/MSI high tumors selectively demonstrate upregulated expression of multiple immune checkpoints to offset the activated immune response and PD-L1 expression, as a regulatory mechanism has been shown to be correlated with dMMR/MSI high in multiple cancer types (49). The correlation between high TMB and MSI-high is, however, variable between tumor types with concordance high in gastrointestinal cancers but low in lung cancer and melanoma (51). The majority of dMMR or MSI-high tumors have a high TMB; however, only a small number (16%) of tumors with high TMB are MSI-high across multiple different cancer types (51).

The pituitary cases reported thus far do not support the use of established biomarkers utilized in other cancer types. The patient we have described responded to dual ICI therapy despite being mismatch repair proficient, having a low mutation burden and undetectable PD-L1. Lin et al. reported significant response to dual ICI therapy in a patient with increased TMB and MSH6 mutation and proposed that TMZ-induced hypermutation may increase tumor response to ICIs. In contrast, Caccese et al. reported no response to single agent anti-PD-1 therapy in a patient with MSH2 and MSH6 mutation who was expected to respond given the association with dMMR, TMB, and response to ICI therapy in other cancers. These cases highlight the need for further research to identify biomarkers for response in pituitary disease and suggest that patients with aggressive disease who have failed to respond to standard therapy should not be excluded based on a lack of currently established biomarkers on tumor analysis.

### Timing of Immunotherapy in Relation to Temozolomide

TMZ is well established as first line chemotherapy for APTs (52). As described above, high TMB may predict response to ICI. It is on this basis, Lin et al. proposed the potential that a TMZ induced hypermutation may increase pituitary tumor response to ICI. However, TMZ may have other effects on the tumor immune environment that may potentially decrease the response to ICIs. Furthermore, a number of studies in different cancers have reported improved response to systemic chemotherapy *following* immunotherapy (53–58). Although there is no similar clinical data for pituitary tumors, pre-clinical studies in murine glioblastoma models suggest that TMZ may impair response to immune checkpoint blockade. TMZ causes systemic immunosuppression, depletion of tumor infiltrating lymphocytes and inhibits JAK/STAT pathway signaling which decreases PD-L1 expression and may limit the effect of PD-1/PD-L1 checkpoint inhibitors in the treatment of these tumors (59–61). In murine glioblastoma models, systemic TMZ was inferior to locally administered TMZ in combination with anti-PD-1 due to the immunosuppressive effects of systemic TMZ (60). Some effects of TMZ on the immune microenvironment seem to be dose related. Standard compared with protracted low dose TMZ dosing causes an upregulation of gene signatures of T cell exhaustion and inhibitory checkpoint markers (62). PD-1 monotherapy for murine glioma models is associated with increased survival which is negated by the addition of standard dose TMZ therapy while being preserved with addition of the lower dose regimen (62). The effects of TMZ on the immune microenvironment in pituitary tumors, the interaction with ICI treatment, and consideration of timing of ICI and TMZ require further investigation.

### Vascular Endothelial Growth Factor Inhibition Therapy and Pituitary Tumors

The VEGF signaling pathway has been implicated in the tumorigenesis of many cancer types. It has a physiological and pathological role in angiogenesis and vascular permeability as well as modulating the immune microenvironment *via* several mechanisms which promote a pro-tumor immunosuppressive microenvironment (12, 63, 64). VEGF targeted therapies including antibody mediated inhibition of VEGF and VEGF receptor tyrosine kinase inhibitors are now used successfully in the treatment of many cancers (64). In pituitary tumors, markers of angiogenesis such as VEGF expression and vascular density are increased in APTs compared with non-APTs; however, the significance of this with respect to anti-VEGF treatment response is uncertain (65–69). Several potential biomarkers such as VEGF expression have been investigated in other cancers with inconclusive findings, and there are currently no validated biomarkers for response to VEGF inhibition (VEGFi) therapy (70–75).

Clinical experience with VEGFi therapy for the treatment of APTs has been limited but promising. Thirteen cases of APT or PC treated with VEGFi therapy have been described, ten of which responded to treatment (2, 7–10). Nine of these were treated with bevacizumab, four in combination with TMZ, and five following unsuccessful treatment with TMZ (2, 7–9, 11). One case has been reported of response to the VEGF-2 inhibitor apatinib in combination with TMZ (10). Of the cases which progressed, two were treated with bevacizumab and one with the VEGF receptor inhibitor sunitinib (2).

### Combination Immune Checkpoint Inhibitor and Vascular Endothelial Growth Factor Inhibition Therapy

There are no cases reported previously of the use of ICI and VEGFi therapy in the same patient for the management of an APT. In our case, a good response to ICI therapy with subsequent progression was followed by a stable response to VEGFi therapy. Whether an improved response to VEGF inhibitor therapy could have been seen if used prior to or concomitant with ICI therapy is not clear. However, a rationale for combination therapy has been established in other cancer types. Tumor angiogenesis contributes to an immunosuppressive microenvironment by decreasing the abundance and function of tumor infiltrating lymphocytes, increasing markers of T cell exhaustion and increasing the abundance of pro-tumor Treg lymphocytes (76). Targeting angiogenesis with anti-VEGF therapies converts the immunosuppressive tumor microenvironment to an immunosupportive one which in turn promotes the effect of ICIs (76, 77). A number of clinical trials have examined the efficacy of combination ICI and VEGFi therapy in melanoma, renal cell carcinoma, and non-small cell lung cancer with favorable results, demonstrating improved response and survival for combination therapy when compared directly and indirectly with treatment regimens consisting of single agent ICI or anti-VEGF therapy (78–80). Currently, in the management of APT, the effectiveness of ICI and VEGF inhibition therapy as monotherapies still needs to be established; however, consideration of timing of ICI and VEGF therapy may be important and should be investigated further.

### Adverse Effects of Novel Therapies for Aggressive Pituitary Tumors

The use of ICIs may be limited by the occurrence of immune related adverse events (irAEs) which can occur in up to 60% of patients treated with anti-CTLA4 antibodies and up to 20% of patients treated with anti-PD-1 and anti-PD-L1 antibodies (81). Fatal irAEs occur in 0.3–1.3% of treated patients and tend to occur early in the course of treatment (82). The most common irAEs are skin rash and colitis and less commonly include hepatitis, nephritis, pneumonitis, pancreatitis, myocarditis, episcleritis, uveitis, and a number of endocrinopathies and neuropathies. The time to onset and severity of irAEs depend on the type of irAE as well as the dose and class of ICI administered. Anti-CTLA antibodies have a higher incidence of irAEs than anti-PD-1 and anti-PD-L1 antibodies, and combination ICI therapy is associated with the highest incidence of irAEs. The mainstay of treatment of irAEs is immunosuppression with corticosteroids or other immunosuppressive agents (81, 83–85). ICIs should be withheld and reintroduced following resolution of the irAE considered on an individual basis although in the case of certain severe irAEs, rechallenge is not recommended (81). The risk of developing irAEs is difficult to predict in patients receiving ICI therapy, and there are no formal recommended prevention strategies. Patients receiving ICI therapy should undergo regular surveillance for irAEs (81).

Adverse effects of VEGFi occur as a result of endothelial dysfunction and include hypertension in up to 32% of patients and proteinuria in 23% of patients, as well as venous and arterial thromboembolic events, cardiotoxicity, impaired wound healing, gastrointestinal perforation, and increased risk of hemorrhagic events (86, 87).

There is emerging experience using the combination of ICI and VEGFi therapy. Seminal phase III trials of atezolizumab plus bevacizumab in non-small cell lung cancer and hepatocellular carcinoma did not reveal any new safety signals (80, 88). Reassuringly, the safety profile of the combination appeared to be consistent with the safety profile of the individual medications (80).

When considering the use of ICIs or VEGFi in the management of APTs, judicious risk assessment is paramount, taking into account the limited clinical experience thus far in APTs, the potential but unproven efficacy of these drugs, and the risk of adverse effects.

## Conclusion

The case we have reported demonstrates excellent initial response of a pituitary carcinoma to combination anti-CTLA4 and anti-PD-1 ICI therapy despite exhibiting an absence of biomarkers considered predictive of response. In this case, ICI therapy undoubtedly prolonged survival and reduced morbidity in this patient. Our experience and that of the previously published pituitary cases suggest that the combination of CTLA4 and PD-1 blockade but not PD-1 blockade alone may be more effective in the treatment of APT and PC and a rationale for this has been described. In addition, the case raises the possibility of sequential or combination ICI and VEGFi therapy as novel therapy for APTs. These new approaches to treatment of APTs represent promising new avenues for research.

## Data Availability Statement

All datasets presented in this study are included in the article/supplementary material.

## Ethics Statement

Written informed consent was obtained from the individual(s) for the publication of any potentially identifiable images or data included in this article.

## Author Contributions

All authors listed have made a substantial, direct, and intellectual contribution to the work and approved it for publication.

## Funding

LL is supported by a University Postgraduate Award from the University of New South Wales.

## Conflict of Interest

The authors declare that the research was conducted in the absence of any commercial or financial relationships that could be construed as a potential conflict of interest.
